# A behavioral strategy to minimize air pollution exposure in pregnant women: a randomized controlled trial

**DOI:** 10.1186/s12199-017-0633-8

**Published:** 2017-04-04

**Authors:** Marzieh Araban, Sedigheh Sadat Tavafian, Saeed Motesaddi Zarandi, Ali Reza Hidarnia, Andrea Burri, Ali Montazeri

**Affiliations:** 1 Social Determinants of Health Research Center, Ahvaz Jundishapur University of Medical Sciences, Ahvaz, Iran; 2 Department of Health Education, Faculty of Medical Sciences, Tarbiat Modares University, Tehran, Iran; 3 Department of Environmental Health Engineering, Faculty of Health, Shaheed Beheshti University of Medical Sciences, Tehran, Iran; 4 Institute of Psychology, University of Zurich, Zurich, Switzerland; 5 Mental Health Research Group, Health Metrics Research Center, Iranian Institute for Health Sciences Research, ACECR, Tehran, Iran; 6 Faculty of Humanity Sciences, University of Culture & Science, ACECR, Tehran, Iran

**Keywords:** Air pollution, Pregnancy, Behavior, Transtheoretical model, Randomized controlled trial

## Abstract

**Background:**

Pregnant women and their fetus belong to a sensitive group in response to air pollution hazards. The aim of this study was to evaluate the effectiveness of a theory-based educational program to change pollution exposure behavior in pregnant women.

**Methods:**

In this randomized controlled trial, pregnant women attending a prenatal clinic in Tehran, Iran were selected and randomized into the experimental and control groups. The inclusion criteria were age between 18 and 35 years, having a history of pregnancies without adverse outcomes and not suffering from chronic diseases. Data collected at baseline and 2-month follow-up. At baseline face-to-face interviews were conducted using a valid and reliable questionnaire including items on demographic characteristics, stages of change, self-efficacy, decisional balance and practice regarding air pollution preventive behaviors. The intervention composed of three parts: motivational interviewing, a booklet and daily small message service (SMS). The control group received no intervention except receiving usual care. Follow-up data were collected after the intervention. Data were analyzed performing t-test, Mann–Whitney U test, and chi-squared.

**Results:**

In all the data for 104 pregnant women (53 in the intervention and 51 in the control group) were analyzed. The mean age of participants was 27.2 (SD = 4.11) years and it was 22.89 (SD = 8.75) weeks for gestational age. At baseline there were no significant statistical differences between intervention and control groups on the study measures while we found significant group differences in terms of stages of change, self-efficacy, perceived benefits and practice regarding air pollution preventive behaviors at follow-up assessment (*P* < 0.05).

**Conclusion:**

The findings indicated that the TTM-based intervention was effective in increasing air pollution preventive behaviors among pregnant women. This study provided a framework to modify some psychosocial determinants of air pollution preventive behavior other than knowledge using constructs of Transtheoretical model of behavior change, additionally results suggests the importance of education and makes enlightenment of the air pollution risk knowledge accelerate.

**Trial registration:**

IRCT2012091010804N1

## Background

Air pollution has severe impact on population’s health, with both short- and long-term consequences [1–3]. Different groups of individuals are affected by air pollution in different ways. Pregnant women and their fetuses, for example, belong to a very vulnerable group and are particularly susceptible to the adverse effects of hazardous air pollutants [4]. Some of the adverse outcomes of exposure to outdoor air pollution for pregnant women include low birth weight of the baby, preterm labor, and intrauterine growth retardation that in turn can lead to morbidity and mortality in later life [3, 5–7]. Such adverse effects further cause a substantial financial burden for society due to increased costs imposed on the health care system [8]. National Surveys of Air Pollution suggest that Tehran (the capital of Iran) is one of the most polluted cities in the world [9, 10]. Despite the strict application of policies and regulations to control the problem, a recent study suggests that current levels of air pollutants in Tehran are above healthy levels [9].

Evidence suggests that the developments of new interventional and prevention framework that focus not only on community-level regulations but also include individual-level perspectives are necessary and could potentially offer a new solution to the problem [11].

Although the link between exposure to air pollution and adverse health outcome are well documented, proper air pollution self-care behaviors guidelines are lacking [12], few studies recommended some preventive behaviors including avoiding going outside, wearing mask [11, 13] and keeping windows closed. Among all recommended preventive behaviors, avoiding going outside is more emphasized [11–13].

There is also data indicating that the effects of exposure to air pollution could be reduced for particularly vulnerable groups, such as pregnant women, with help of certain behavioral prevention strategies (on an individual level), such as reducing the time spent outdoors [14, 15].

To achieve maximum effectiveness, preventive strategies and interventions should be theory-driven or should at least target the construct, to increase the likelihood of behavioral changes toward the desired outcomes [16]. As such the Transtheoretical Model (TTM) is a widely applied theory in the studies of behavioral changes allowing researchers to categorize people based on their openness to change and to foster our understanding regarding the psychosocial differences between those individuals who do perform a particular behavior and those who do not [17]. It assesses an individual's readiness to act on a new healthier behavior, and provides strategies, or processes of change to guide the individual through the stages of change. The model consists of four constructs: stages of change, decisional balance, self-efficacy and processes of change [18]:

- 1. The ‘stages of change’ places people in one of five stages of readiness/openness to engage in a given behavior. These are: precontemplation, contemplation, preparation, action, and maintenance. The proposed stages of change indicate that individuals who are changing their behavior move through a series of stages. Precontemplation is the stage in which individuals do not intend to change their behavior in the near future. In contemplation, individuals are considering it but are not committed to it and in preparation; individuals are committing to and planning for imminent change. Action is the stage in which behavior has changed and maintenance is the stage in which people are working to prevent relapse [18]. Transition between the different stages depends on an individual's motivation and self-efficacy levels, which can be enhanced by the use of cognitive and behavioral processes such as consciousness raising, dramatic relief and helping relationship [19].
- 2. Decisional balance reflects the individual’s relative weighing of the pros (or benefits) and cons (or barriers) of changing and has become critical constructs in the model and is one of the best predictors of future change [20].
- 3. Self-efficacy refers to a person's sense of confidence in his or her ability to perform a particular behavior [10, 21] and has been adapted from Bandura's self-efficacy theory [22]. A change in the level of self-efficacy can predict a change in behavior if adequate incentives are present.
- 4. Processes of change are 10 processes of change are “covert and overt activities that people use to progress through the stages” [17]. To progress through the early stages, people apply cognitive, affective and evaluative processes. As people move toward Action and Maintenance, they rely more on commitments, conditioning, contingencies, environmental controls, and support.

To the best of our knowledge, no study has investigated behavioral change in terms of prevention of air pollution exposure in pregnant women. Such a study is needed, given the importance and relevance of this topic in a critically polluted environments such as Tehran,. As metioned before this becomes more crucial considering that it has serious adverse health effects at population level and particularly vulnerable groups such as pregnant women and their fetuses. The objective of this study was to evaluate the effectiveness of a TTM-based educational intervention to persuade changes in exposure behavior of pregnant women in response to air pollution and to eventually promote health in this population subgroup.

## Methods

### Design

This was a parrall group randomized controlled trial to assess effectivness of a theory-based intervention to minimize air pollution exposure among pregnant women.

### Participants

In all 135 women were contacted, but five women (3.5%) refused to participate. No differences in socio-demographic variables between women refusing to participate and women interested in participating could be detected. Out of the 130 interested potential participants 110 women met the eligibility criteria. These criteria included: aged 18 to 35 years, having a history of pregnancy without any adverse outcomes (e.g. Preterm labor); not suffering from any chronic disease (e.g. diabetes, hypertension, kidney of cardiovascular problems), no previous history of infertility, gestational age between 20–24 weeks, and having owning a mobile phone for receipt of daily messages. Women were excluded from the study if they were experiencing complications during their pregnancy such as bleeding, hypertension, or any other complication resulting in permanent bed rest or hospitalization.

### Study setting and procedure

The study was conducted between June 2012 and September 2012 at the prenatal care ward of a teaching hospital in Tehran, Iran. The center is one of the largest obstetrics hospitals in Tehran. Women attending the hospital were approached by their visiting midwife and after declaring their interest to participate in the study, they received detailed information regarding the aim of the study and were asked to provide informed consent. Then participants were randomly assigned to either the control or the intervention group according to simple randomization procedures (computerized random numbers). After data collection at 2- month follow-up, women in both the control and the intervention group were given a doll as a thank you gift. Participants by pen and paper completed questionnaires at clinic.

### Intervention

The educational intervention consisted of three components including 1. A one-hour motivational interviewing session conducted by principal investigator focusing on preventive behaviors to air pollution exposure, 2. Daily small message service (SMS) over a one-month span. 3. An educational booklet regarding air pollution. The goal of the intervention was to transit the participants from the precontemplation stage into the action stage of the TTM. Motivational interviewing sessions were held in small groups of six participants and aimed at helping the participants expressing their own reasons for and against change and how their current behavior or health status affects their ability to achieve their life goals or fulfill core values. Key motivational interviewing methods used in the intervention included asking open-ended questions (e.g., what are benefits or barriers to minimizing exposure to air pollution?), affirming (to enhance self-efficacy) and supporting the participants during the session (e.g., it is great that you manage your time so that you not to go outdoors in the peak hours of air pollution in the morning or evening, and ensuring participant that SMS will be sent to them in order to informing participants of air pollution levels). During these sessions, participants were asked to report about their own current behaviors and the reasons for such behaviors. Together with the interviewer, these behaviors were analyzed and discussed and participants were motivated to change their unhealthy behaviors, full description about this part has been published elsewhere [23]. At the end of the sessions, each participant was provided with an educational booklet containing information about air quality in Tehran, the health risks of air pollution for both mother and fetus during pregnancy, motivating illustrations and pictures, and short statements to encourage participants to engage in preventive behaviors. Daily SMS was sent to the participants to inform them about Tehran’s daily air quality and to encourage participants to behave as recommended in the educational sessions. The SMS, as well as information regarding the air quality, were provided by Tehran Air Quality Control Company (AQCC). The control group received neither motivational interviews nor any detailed information regarding air quality in Tehran but only routine prenatal care. No interventions were performed for the control group. Yet, the data of air quality status could be found on Tehran Air Quality Control Company website that is available to public at: http://air.tehran.ir/.

### Measures

- i) Demographic and obstetric information: A basic demographic characteristics assessing women’s age, educational status (also of their husband), monthly family income, and pregnancy characteristics such as gestational age and pregnancy order was completed at baseline.
- ii) Stages of behavioral changes regarding prevention of exposure to air pollution: This part was constructed based on the Prochaska’s stage of change [17] and consisted of five statements according to which participants were stratified into different stages of change: precontemplation (1), contemplation (2), preparation (3), action (4) and maintenance (5). The participants were asked to respond to one question choosing the statement that best described their status. Response options were: (1) “I am currently not engaging in any preventive behaviors regarding air pollution exposure such as staying indoors in the daily hours (from 7 to 9 am and from 6 to 9 pm) or on days where the air quality is critical and I further do not enter high traffic areas of the city and I am not thinking to do so in the upcoming six months”. “I am currently not doing any of the behaviors but I am thinking of doing so in the upcoming six months” (2) “I am currently not doing any of the behaviors but I plan to do so within the next month” (3) “I am currently doing the behaviors but I have been doing so for less than six months” (4) “I am currently doing the behaviors” (5) “I have been doing them already for more than six months”.
- iii) Self efficacy: The following questions were used to assess self-efficacy (i.e. confidence in one’s ability to perform preventive behaviors regarding exposure to air pollution): (1) I can stay indoors in the peak hours of the air pollution - from 7.00 o'clock to 9.00 o'clock in the morning, (2) I can stay indoors in the peak hours of the air pollution - from 6.00 o'clock to 9.00 o’ clock in the evening. (3) I can stay home on days that air quality is in the crisis situation, (4) I can avoid entering into the high traffic area of the city. Each item was responded to on a 4-point Likert-type scale (from not at all sure = 1 to completely sure about recommended preventive behaviors = 4). The total score ranged from 4 to 16 with higher values indicating better self-efficacy. These questions were developed and validated in a previous study conducted by the authors [23] where they showed the excellent validity for both CVR and CVI (CVR = 1 and CVI = 1) and good internal consistency (α = 0.74).
- iv) Decisional balance: It includes items on perceived benefits and barriers to air pollution preventive behaviors. Perceived benefit was assessed with 7 items, each rated on a 5-point Likert-type scale ranging from ‘strongly agree’ (5) strongly ‘disagree’ (1). Perceived barrier was assessed with 5 items. The total score for the perceived benefits ranged from 7 to 35 and for perceived barriers from 5 to 25. The content validity as assessed by content validity index was 0.89 for benefits and 0.83 for barriers. The Cronbach’s alpha were α = 0.79 and α = 0.91 for benefits and barriers, respectively.
- v) Preventive behavior in terms of air pollution exposure during the past month: This part included the following four items: 1. How often did you stay indoors in the peak hours of the air pollution - from 7 to 9 am? 2. How often did you stay indoors in the peak hours of the air pollution - from 6 to 9 pm? 3. How often did you stay indoors in the days that air quality is in the crisis situation? 4. How often did you avoid entering into the high traffic area of the city? Each item was rated on a 5-point scale ranging from ’always‘ (5) to ’never’(1). Total scores ranged from 5 to 20, with higher values indicating more preventive behavior. The content validity as assessed by content validity index was 0.89. The Cronbach’s alpha was α = 0.84.

### Sample size

To detect an increase in self-efficacy as the best predictor of behavioral change of around 3.5 [23] with a two-sided 5% significance level and a power of 80%, a sample size of 55 participants per group was necessary given an anticipated 10% dropout.

### Randomization sequence

Participants assigned to comparison groups based on random allocation where we first generated.

### Randomization type

Participants were randomly assigned following simple randomization procedures to intervention or control groups.

### Allocation concealment

The intervention program implemented for women according to the randomization schedule. Each pregnant woman was assigned an order number. Then the odd numbers were selected to enroll in the intervention group.

### Statistical analysis

All data analyses were conducted using Statistical Package for the Social Sciences (SPSS) version 15.0 (SPSS Inc., Chicago, IL, USA). Differences in sociodemographic and obstetric characteristics between the control and intervention group were assessed using a t-test for continuous variables and the chi-squared test for binary/categorical variables and proportions. Mean scores between the two groups were compared using an independent t-test or a Mann Whitney U test where necessary. An alpha error of < 0.05 indicated statistical significance.

### Ethics

The study was approved by the Ethics Committee of Tabiat Modares University. All participant asked for informed consent.

## Results

### Study sample

**Fig. 1:**
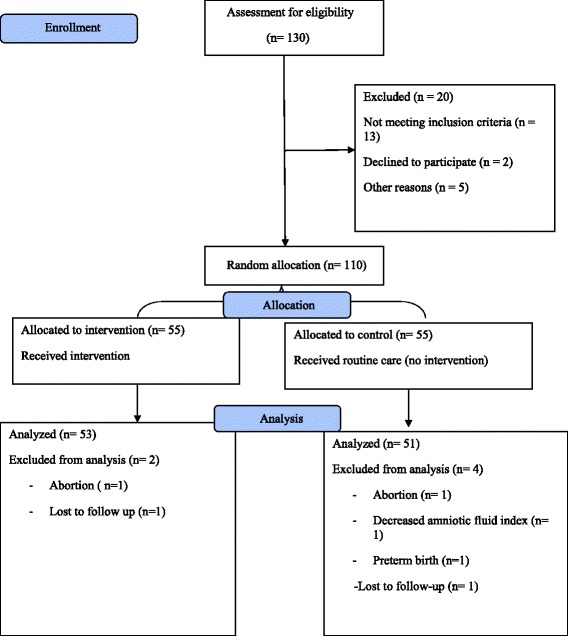
Flow diagram of the study

In all 104 pregnant women were included in the final analyses. Of the *n* = 55 participants allocated to either the intervention or control group, *n* = 2 (3.6%) and *n* = 4 (7.2%) dropped out in the 1-month follow-up. The reasons for the study drop-out are described in Fig. 1.

The sociodemographic and obstetric characteristics of the overall sample and according to group assignment are shown in Table 1. The mean age of the participants was 27.1 years (SD = 4.1), ranging from 20 to 35 years. Mean gestational age was 22.9 weeks (SD = 8.75).

**Table 1:** The characteristics of participants

	All	Intervention (*N* = 53)		Control (*N* = 51)		
		Mean (SD)	No. (%)	Mean (SD)	No. (%)	P
Age	27.11 (4.10)	27.37 (3.96)		27.02 (4.96)		0.67^a^
Gestational age	22.92 (8.09)	23.48 (8.83)		22.29 (8.79)		0.49^a^
Pregnancy order						0.82^a^
The first	77 (71.6)		40 (75.5)		38 (72.5)	
The second or more	26 (28.4)		13 (24.5)		14 (27.5)	
Education						0.42^b^
≤Secondary	65 (62.6)		31 (58.5)		34 (66.7)	
> Higher	39 (37.4)		22 (41.5)		17 (33.3)	
Husband's education						0.15^b^
≤ Secondary	68 (65.4)		31 (58.5)		37 (72.5)	
> Higher	36 (34.5)		22 (41.5)		14 (27.5)	
Income						0.41^b^
Poor	15 (14.5)		6 (11.3)		9 (17.6)	
Fair/good	89 (85.5)		47 (88.7)		42 (82.4)	

^a^Derived from t-test

^b^Derived from chi-square

### Findings

At baseline, no statistically significant differences between the control and the intervention group in terms of stages of change (all participants were in the precontemplation), perceived benefits (*p* = 0. 54) and barriers (*p* = 0.24) and self-efficacy (*p* = 0. 06) could be detected. After the intervention, however, stage of change, perceived benefits and self-efficacy differed significantly between the the two groups, with all of the three variables showing a significant increase in the intervention group (*p* < 0.001 for all three constructs) but not in the control group, respectively (Tables 2 and 3). Additionally, results obtained from the Mann Whitney U test showed that the mean rank of practice was significantly higher in the intervention group compared to the control group.

**Table 2:** Stages of change at 1- month follow up

	Intervention (*N* = 53)	Control (*N* = 51)	
	No. (%)	No. (%)	P^a^
Pre-action	7 (13.2)	48 (94.1)	
Action	46 (86.8)	3 (5.9)	
			<0.001

^a^ Derived from chi-square

**Table 3:** Comparisons of perceived benefits, perceived barriers, self-efficacy and practice between two groups

	Before intervention			After intervention		
	Intervention (*N* = 55)	Control (*N* = 55)		Intervention (*N* = 53)	Control (*N* = 51)	
	Mean (SD)	Mean (SD)	P^a^	Mean (SD)	Mean (SD)	P^a^
Perceived benefits	30.46 (3.97)	30.02 (3.66)	054	32.5 (2.62)	29.7 (2.65)	<0.001
Perceived barriers	11.7 (5.10)	12.9 (5.3)	0.24	7.42 (2.21)	10.3 (3.13)	<0.001
Self-efficacy	11.92 (2.93)	13.08 (2.64)	0.056	15.2 (1.45)	12.7 (2.12)	<0.001
Practice	11.2 (2.1)	11.5 (2.6)	0.73	19.4 (1.75)	10.6 (2.1)	<0.001

^a^Derived from t-test

The within-group responses to the intervention were assessed by calculating the changes in the measures from pre-test to post-test, with positive values indicating an increase, and negative values indicating a decrease. Group comparison showed that the differences of perceived benefits and self-efficacy were significant at a *p* < 0.05 level. No significant change could be detected for perceived barriers (*p* > 0.05; Table 4).

**Table 4:** Comparison of score changes between two groups

	Intervention (*N* = 53)	Control (*N* = 51)	
	Mean change^a^ (SD)	Mean change^a^ (SD)	P^b^
Perceived benefits	2.04 (4.16)	0.272 (3.67)	0.003
Perceived barriers	−4.42 (5.02)	−2.61 (5.51)	0.10
Self-efficacy	3.35 (3.34)	−0.22 (2.5)	0.0001

^a^Follow-up score minus baseline score

^b^Derive from Mann–Whitney U test

## Discussion

Our study findings suggest that TTM-based educational interventions can increase preventive behaviors aimed at reducing exposure to pollution exposure. Overall, women in the intervention group performed the suggested preventive behaviors better and more often (reduce exposure time to hazardous outdoor air pollutants) than the control group. The results further showed that focusing on self-efficacy and own beliefs to change helped women in the intervention group to perform the desired behaviors. This is consistent with the findings from Bazargan and Downer and colleagues whose study results suggested that improving people’s self-efficacy via motivational interventions and sending text messages could lead to an increase in preventive health behaviors [20].

In line with our study Karatay et al., found that motivational interviewing could change pregnant women’s perception regarding the importance of behavioral changes and enable them to eliminate unhealthy behaviors in pregnancy [24] while another study conducted by Hayes et al., did not support the effectiveness of motivational interviewing in changing towards healthy behavior in pregnant women [25]. One possible explanation for such differences might be due to the fact that they have assessed different types of behaviors. For instance, while air pollution preventive behavior needs more time management, smoking preventive behaviors needs more complex psychological process.

Our findings are in accordance with previous study results of theory-based health behavior changes for pregnant women [26, 27]. Aveyard et al., investigated the stages of smoking cessation behavior in a sample of pregnant women in England and concluded that TTM-based intervevtions are more effective than interventions which use only the stages of chang construct [26]. Results from another study conducted by Lawrence and colleagues showed that quittng smoking in pregnancy could be increased using TTM-based approaches [27].

In the present study, the best interventional effects were observed in the constructs “perceived benefits” and “self-efficacy”. This is in line with reports from previous studies. where TTM-based educational intervention significantly enhanced the stages of behavioral change and increased perceived benefits and self-efficacy [28, 29]. These findings could be better understood when bearing in mind the strong and weak points of the TTM [17, 30]; Progression from precontemplation to the stage of action leads to a one SD increase in perceived benefit (pros) whereas it also leads to a one-half SD decrease in perceived barriers (cons).

Although reducing exposure to air pollution protect pregnant women' health, staying indoor may interfere with needing to go to work, getting children to school or on the bus or needing to do household jobs and shopping. Perhaps pregnant women may plan to do their duties on hours which air pollution is not critical to protect their health and their growing baby.

A previous study showed that although the knowledge regarding air pollution health risk seems to be desirable, they did not comply preventive behaviors [31]. On the other hand, just being knowledgeable about something does not trigger doing desired action. Since determinants of behavior are unlimited [22, 32], we should go beyond knowledge and should change attitude, beliefs and other important determinants of behavior.

Long-term environmental health education programs at the level of population are needed to change attitude and behaviors regarding air pollution of Tehran. In this study we did not explore the social impact of education that might be the subject of future studies.

In the one-month follow-up, three participants from the control group were excluded from the study due to pregnancy complications as opposed to one person in the intervention group. Although this difference is not statistically significant, it might be clinically important feature that could be assessed as a hypothesis in future researches.

## Limitation

As in every study, there are several limitations that need to be considered. Because of the small sample size, we had to collapse stages into two categories. This has been successfully used in other studies with small sample size [19].

## Conclusion

Educational intervention strategies based on Transtheoretical model can increase preventive behaviors in pregnant women aimed at decreasing exposure to air pollution. The study results emphasize the effectiveness of such an approach and its mechanisms that contribute to its effectiveness as well as highlights its potential for future educational interventions aimed at increasing air pollution preventive behaviors in vulnerable groups such as pregnant women. Moreover, the current research provided a plan for evidence-based decision-making regarding air pollution risk communication and behavior change. Therefore, health authorities might use it.

## Abbreviations

CVI: Content validity index
CVR: Content validity ratio
